# Redox Biomarkers Assessment after Oral Administration of Wine Extract and Grape Stem Extract in Rats and Mice

**DOI:** 10.3390/molecules28041574

**Published:** 2023-02-06

**Authors:** Fotios Tekos, Zoi Skaperda, Periklis Vardakas, Despina Kyriazi, Georgios C. Maravelis, Konstantinos Poulas, Ioannis A. Taitzoglou, Charitini Nepka, Demetrios Kouretas

**Affiliations:** 1 Department of Biochemistry and Biotechnology, University of Thessaly, 41500 Larissa, Greece; 2 Department of Pharmacy, University of Patras, 26504 Patras, Greece; 3 Department of Physiology, Faculty of Veterinary Medicine, School of Health Sciences, Aristotle University, 54124 Thessaloniki, Greece; 4 Department of Pathology, University Hospital of Larissa, 41110 Larissa, Greece

**Keywords:** antioxidants, polyphenols, redox biomarkers, toxicological study, wine extract

## Abstract

Wine and by-products of the winemaking process, such as grape stems, are rich in bioactive polyphenolic compounds that might be beneficial for animal and human health. In recent years, the administration of dietary polyphenols with strong antioxidant and cytoprotective properties has constituted an emerging line of research interest toward disease prevention. However, in scientific literature, only a limited number of studies have investigated the safety and the toxicological risks of polyphenolic compounds in vivo. Based on the above, the purpose of the present study was two-fold: first, to examine the effects of oral administration of a grape stem extract, derived from the Greek red wine Mavrodaphne, on mice redox biomarkers; and second, to investigate the biological effects of oral administration of a wine extract, derived from the emblematic Greek red wine Xinomavro, on rats. Toward this purpose, body weight, growth rate, hematological, biochemical, and histopathological parameters, as well as a panel of redox biomarkers, were examined. According to our results, the administration of Mavrodaphne grape stem extract in mice induced alterations in redox homeostasis, preventing mice from the adverse effects of lipid peroxidation. Contrariwise, the administration of Xinomavro wine extract induced both beneficial and harmful outcomes on rat redox status determined by the examined tissue. Collectively, our study reports that the Mavrodaphne grape stem extract, a serious pollutant when disposed in environmental matrices, is an important source of bioactive polyphenolic compounds that could protect from oxidative damage and improve animal and human health. Finally, the Xinomavro wine extract exerts tissue-specific changes in redox balance, which are indicative of the complexity that characterizes the biological systems.

## 1. Introduction

Polyphenols comprise a large group of bioactive phytochemical compounds with potent antioxidant and cytoprotective properties that are present in large quantities in plant-based foods [1,2,3,4,5,6,7,8,9,10,11,12,13]. In the scientific literature, a multitude of in vitro and in vivo studies have stressed their beneficial properties for human health. More specifically, previous publications have clearly demonstrated that polyphenols possess antioxidant, anti-inflammatory, anti-allergic, and vasodilatory properties [14,15], providing strong protection against cardiovascular diseases, arthropathies, neuropathies, and diabetes mellitus [2,10,15,16,17,18,19,20,21,22,23,24,25,26,27,28]. Furthermore, polyphenols participate in the metabolism of lipids and lipoproteins [4,6,29] and are considered as platelet accumulation inhibitors [20,25,30]. In addition, they have anti-tumor functions [12,23,26,28,31,32,33] and exhibit protective effects against sunburn and photoaging by reducing the damage of ultraviolet A (UVA) and ultraviolet B (UVB) radiation on skin [34,35,36,37].

By virtue of the aforementioned properties, polyphenols have attracted considerable scientific interest toward disease prevention, administrated as dietary supplements or through the enrichment of daily diet with polyphenol-rich natural foods. However, their toxicological safety, as well as their polyphenol-induced metabolic alterations, have not been thoroughly described.

A limited number of in vivo studies have examined the toxicological profile and evaluated the safety of polyphenol-rich foods, indicating minimal to no side effects. For instance, the median lethal dose (LD50) of *Gelidium elegans* extract is possibly greater than 5000 mg/kg, and its repeated oral administration in rats for 14 days and for 13 weeks is considered safe. The no-observed-adverse-effect level (NOAEL) has been determined at 2000 mg/kg/day [38]. Furthermore, the oral administration of Clovinol, a polyphenolic extract of clove buds, in rats for 14 days at a dose of 5 g/kg body weight is considered safe. Following a 90-day sub-chronic study of oral administration in rats, the NOAEL was determined at 1000 mg/kg body weight/day [39]. In addition, after a 90-day sub-chronic study of oral administration of purified blueberry polyphenols in ovariectomized Sprague-Dawley rats, the NOAEL was determined at 1000 mg total polyphenols/kg body weight/day [40]. According to a 90-day sub-chronic study of the oral administration of *Vaccinium virgatum* powdered leaves in male and female rats, a dose of 2500 mg/kg body weight is considered safe [41]. Moreover, following a 90-day oral administration of green tea catechins in rats, the NOAEL was determined to be 764 mg/kg body weight/day for males and 820 mg/kg body weight/day for females [42]. In addition, the determination of acute oral toxicity of a grape seed and peel extract in rats showed that the LD50 is higher than 5000 mg/kg [43]. Finally, the sub-chronic oral administration of an extract derived from grape peel and/or grape seeds in rats is considered safe under the specific experimental setup [44,45,46]. The NOAEL for male rats has been determined to be 600 mg/kg/day [44].

Wine, an alcoholic beverage derived from the fermentation process of grape juice, contains a wide variety of water-soluble polyphenols, divided in two main categories: flavonoids, such as flavanols, flavonols, and anthocyanins; and non-flavonoids, such as phthalic acids, tannins, and polishes [16,33]. Red grapes and wines are particularly rich in anthocyanins, accumulated in grape skin, whereas white grapes are mainly rich in flavanols [47]. The longer period of time that the red juice remains in contact with the grape skins and seeds contributes significantly to the higher polyphenolic content of red wines, ranging from 1800 to 3000 mg/L, as compared to the white wines [48].

Xinomavro is an emblematic Greek red wine produced from a red grape variety planted mainly in Central and Northern Greece. The assessment of the antioxidant capacity of a wine extract derived from Xinomavro by a recent study of our research group has demonstrated that the wine extract contains high levels of polyphenolic compounds and exerts strong antioxidant and antimutagenic activities in in vitro cell-free systems [49]. Therefore, the elucidation of its bioactivity at higher levels of complexity, i.e., cell-based and in vivo systems, could provide valuable insights into its potential applications in the food and pharmaceutical industries.

Grape stems, by-products of winemaking process, also contain high concentrations of bioactive phytochemicals. It is worth mentioning that the levels of their polyphenolic compounds are significantly higher than those of the grapes and of the produced wines [50]. However, these by-products have no industrial use and contain high pollution load, thus causing harmful effects, such as mortality, on susceptible organisms in adjacent lakes or rivers [51,52,53]. Their disposal in environmental matrices can cause serious ecotoxicological problems, such as phytotoxicity and eutrophication, degrading the aquatic ecosystems. In Greece, the total amount of grape stems remaining after vinification, is approximately equal to 25,000 tons annually. Hence, the assessment of the safety of polyphenol-rich grape stems for target animal species and their incorporation in animal nutrition could reduce feeding costs, improve animal health status, and contribute to the management of winery industry waste with a significant environmental impact.

Interestingly, previous studies of our research group have investigated the redox-related properties of a grape stem extract derived from Mavrodaphne in in vitro cell-free and cell-based systems [50,54]. Mavrodaphne is an emblematic wine of the Greek vineyard, produced from a red grape variety planted mainly in Northern Peloponnese and on Kefalonia Island. These studies have demonstrated that the grape stem extract possesses potent antioxidant and antimutagenic properties; however, it does not cause any effects on the cellular redox state when administered at non-cytotoxic doses.

Accordingly, the present study has two main objectives; first, to investigate the effects of 28-day oral administration of Mavrodaphne grape stem extract on mice tissue redox biomarkers; and second, to investigate the effects of 14-day oral administration of Xinomavro wine extract on physiological, biochemical, histopathological, and redox parameters in rat blood and tissues. Toward this purpose, the Mavrodaphne grape stem extract was used for the enrichment of a feeding biscuit and administered to mice at a dose of 155.9 mg/kg of body weight/day. On the contrary, the Xinomavro wine extract was administered to rats at a dose of 25 mg of polyphenols/kg of body weight/day, corresponding to a typical human consumption of two glasses of wine/day.

## 2. Results

### 2.1. Effects of Mavrodaphne Grape Stem Extract on Mice Redox Biomarkers

**Figure 1:**
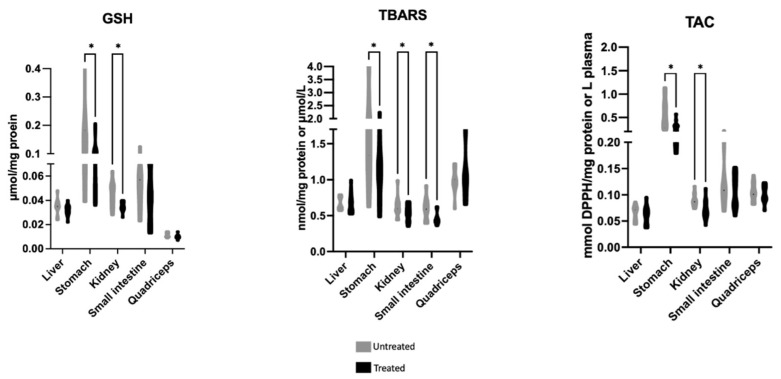
Effects of Mavrodaphne grape stem extract on GSH, TBARS, and TAC levels of mice tissues. *: Statistically significant difference compared to the untreated group (*p* < 0.05).

The effects of Mavrodaphne grape stem extract on mice tissue redox biomarkers are illustrated in Figure 1. The reduced form of glutathione (GSH) and total antioxidant capacity (TAC) levels were statistically decreased in the kidney and stomach of the treated group compared to the untreated group. The thiobarbituric acid reactive substances (TBARS) levels were statistically decreased in the kidney, small intestine, and stomach of the treated group in comparison with the untreated group.

### 2.2. Effects of Xinomavro Wine Extract on Rat Redox Biomarkers

**Figure 2:**
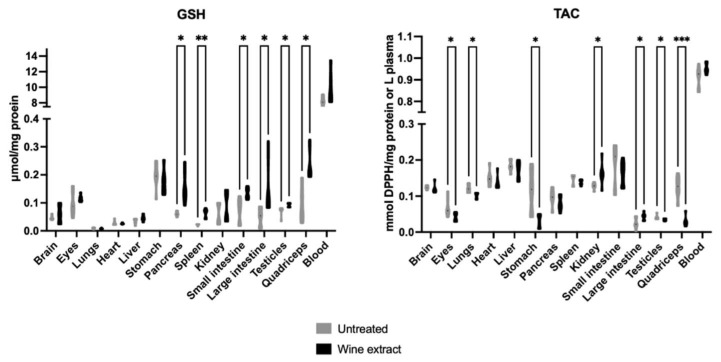
Effects of Xinomavro wine extract on GSH and TAC levels of rat blood and tissues. *: Statistically significant difference compared to the untreated group (*p* < 0.05).**: Statistically significant difference compared to the untreated group (*p* < 0.01), ).***: Statistically significant difference compared to the untreated group (*p* < 0.001).

The administration of Xinomavro wine extract statistically increased GSH levels in the pancreas, spleen, small intestine, large intestine, testicles, and quadriceps of the treated group compared to the untreated group. The TAC levels were statistically decreased in the eyes, lungs, stomach, testicles, and quadriceps and statistically increased in the large intestine and kidney of the treated group compared to the untreated group (Figure 2).

**Figure 3:**
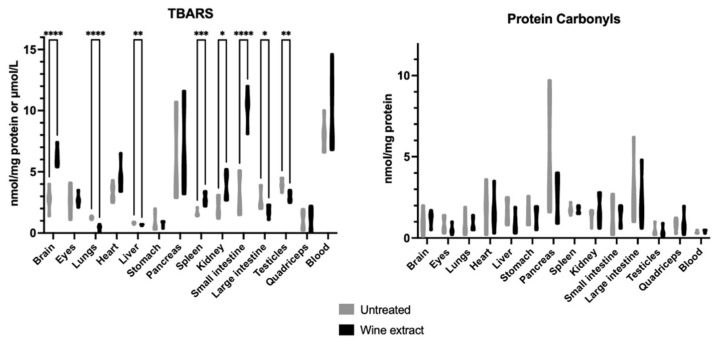
Effects of Xinomavro wine extract on TBARS and protein carbonyl levels of rat blood and tissues. *: Statistically significant difference compared to the untreated group (*p* < 0.05).**: Statistically significant difference compared to the untreated group (*p* < 0.01). ***: Statistically significant difference compared to the untreated group (*p* < 0.001). ****: Statistically significant difference compared to the untreated group (*p* < 0.0001).

The TBARS levels were statistically increased in the brain, spleen, kidney, and small intestine and statistically decreased in the lungs, liver and, testicles of the treated group in comparison with the untreated group. Protein carbonyls were not affected (Figure 3).

**Figure 4:**
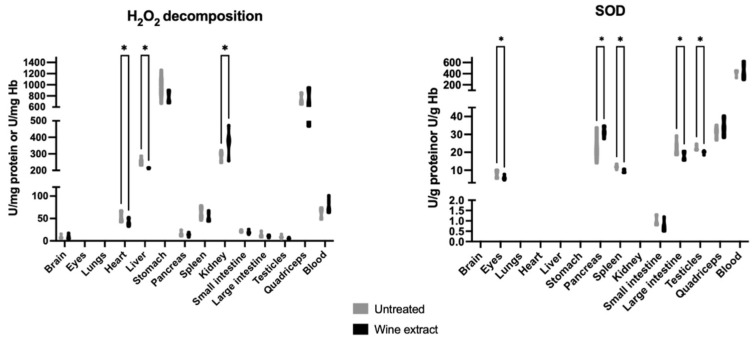
Effects of Xinomavro wine extract on H_2_O_2_ decomposition rate and SOD activity of rat blood and tissues. *: Statistically significant difference compared to the untreated group (*p* < 0.05).

The hydrogen peroxide (H_2_O_2_) decomposition rate was statistically decreased in the heart and liver and statistically increased in the kidney of the treated group compared to the untreated group. The superoxide dismutase (SOD) activity was statistically decreased in the eyes, spleen, large intestine, and testicles and statistically increased in the pancreas of the treated group in comparison with the untreated group (Figure 4).

**Figure 5:**
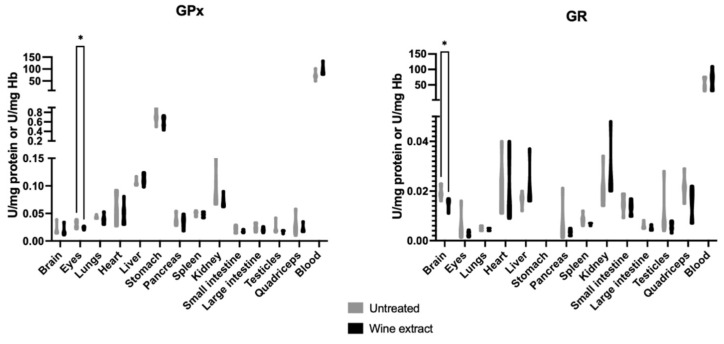
Effects of Xinomavro wine extract on GPx and GR activity of rat blood and tissues. *: Statistically significant difference compared to the untreated group (*p* < 0.05).

The glutathione peroxidase (GPx) activity was statistically decreased in the eyes of the treated group compared to the untreated group. The glutathione reductase (GR) activity was statistically decreased in the brain of the treated group compared to the untreated group (Figure 5).

### 2.3. Effects of Xinomavro Wine Extract on Rat Body Weight and Growth Rate

The daily oral administration of Xinomavro wine extract at a dose of 25 mg of polyphenols/kg body weight (BW) did not cause any adverse effects or mortality during the experimental period. The body weight of the treated group increased from 369.17 ± 22.96 gr on the 1st day to 396.33 ± 23.21 gr on the 14th day. The body weight of the untreated group increased from 370.17 ± 13.53 gr on the 1st day to 397.17 ± 13.66 gr on the 14th day. The growth rate was 1.94 ± 0.12 gr/day for the treated group and 1.93 ± 0.05 gr/day for the untreated group. No significant differences on body weight and growth rate were observed between the treated and untreated groups. In addition, the food and water consumption did not differ in both groups.

### 2.4. Clinical Examination

During the administration of Xinomavro wine extract, daily clinical examination and observation were carried out. No deviations were observed among experimental animals in the same group or between different groups. In addition, during neurological testing on days 1-7 and day 14, no pathological findings were observed in both groups.

### 2.5. Effects of Xinomavro Wine Extract on Rat Hematological and Clinical Chemistry Parameters

The blood results are illustrated in Table 1. The results of the total blood analysis ranged between the normal limits described in the literature for the race, sex, and age of the rats. Furthermore, there were no statistically significant differences between groups.

The results of the clinical chemistry parameters are illustrated in Table 2. The administration of Xinomavro wine extract did not affect the clinical chemistry parameters measured in both groups, and the values ranged between those described bibliographically.

### 2.6. Gross Necropsy

At the end of the study, the rats underwent a detailed post-mortem examination of the internal organs, which showed no differences among size, color, texture, and composition. The content of the hollow organs was normal for all animals in both groups. In addition, the cavities were normal, without any pathological fluid concentration. Hair, skin, mucous membranes, eyes, and external genitalia did not have lesions, as well.

### 2.7. Histopathological Assessment

**Figure 6:**
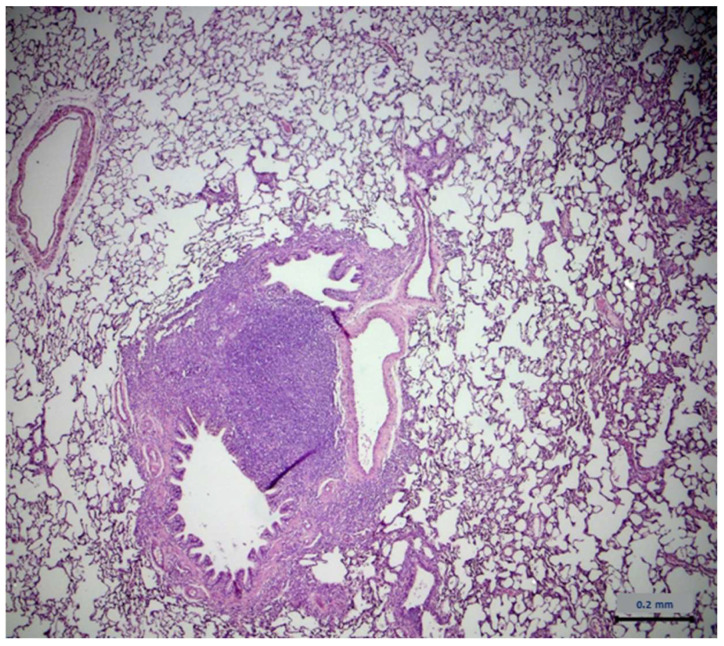
Histology image of untreated rat lung.

**Figure 7:**
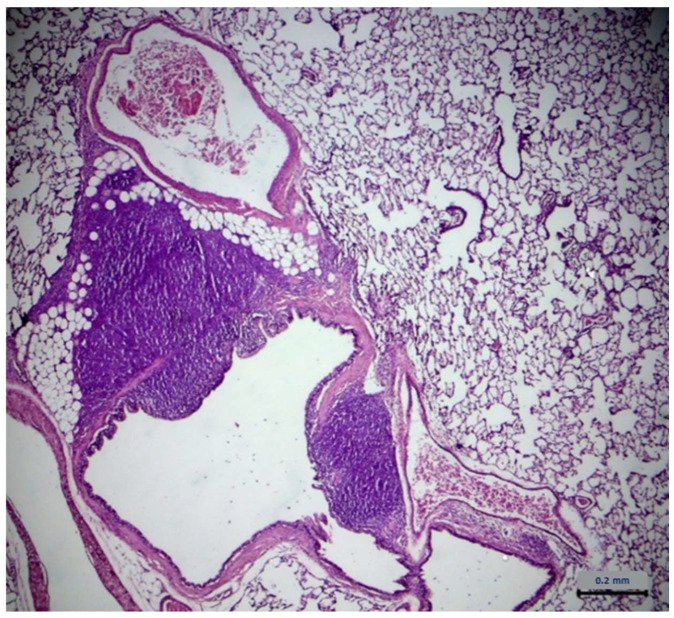
Histology image of treated rat lung.

No lesions related to the administration of Xinomavro wine extract were observed during the histopathological evaluation of the collected organs. However, lesions on the lungs were found in both groups. These lesions were characterized by an increase in perivascular mononuclear cells (mainly lymphocytes), localized around the medium-sized arteries throughout the lungs and extending sometimes to the peribronchiolar areas. In addition, focal infiltrates of mixed inflammatory cells (macrophages, lymphocytes, and occasionally neutrophils) were detected in the median space and in the alveoli. No evidence of infectious agents was found during histological examination. The results are described in Table 3. Table 4 describes the severity of those lesions. The statistical analysis of the data from lung examination showed no difference between groups. Typical lung lesions found in treated and untreated group are shown in Figure 6 and Figure 7, respectively.

## 3. Discussion

This two-fold study examined, first, the effects of 28-day oral administration of Mavrodaphne grape stem extract on mice redox biomarkers and, second, the effects of 14-day oral administration of Xinomavro wine extract on body weight, growth rate, clinical, biochemical, histopathological, and redox parameters in rats. The grape stem extract of Mavrodaphne was administered at a dose of 155.9 mg/kg body weight/day for 28 days. This concentration was based on phytochemical analysis, corresponding to 32 mg polyphenols/kg body weight/day, a dose previously used by Priftis et al. as a safe dose that did not induce any adverse effects [55]. The selected dose for Xinomavro wine extract was 25 mg polyphenol/kg BW/day. The previous dosage scheme was selected as it corresponds to a daily red wine consumption, of the chosen grape variety of 5.1 mL/kg body weight, which is equivalent to the consumption of almost two glasses of wine/day. Wine from the red grape variety Xinomavro was selected to be evaluated as it was observed to have the highest concentration of polyphenolic compounds as compared to other varieties of red and white wines. The duration of the study was 14 days to determine primary conclusions on the safety of the substance, to carry out more extensive studies in the future in accordance with OECD guidelines. None of the tested parameters revealed any significant changes, except for the redox biomarkers.

Particularly, the levels of GSH, the most important endogenous antioxidant molecule, TAC, and TBARS, were determined in mice kidney, quadriceps, small intestine, liver, and stomach following 28 days of administration of Mavrodaphne grape stem extract. The results revealed that the levels of GSH and TAC were decreased in the kidney and stomach of the treated group. Nevertheless, Mavrodaphne grape stem extract acted protectively against lipid peroxidation, as indicated by the decreased TBARS levels in the kidney, small intestine, and stomach. These findings render the grape stem extract from Mavrodaphne as a promising antioxidant agent with protective properties against lipid peroxidation; however, its potential use as an additive of bio-functional animal feed remains under investigation. The paradox obtained in this study lies in the fact that whereas the TAC and GSH levels were decreased, the polyphenolic extract of Mavrodaphne grape stems did also act protectively against lipid peroxidation, as indicated by the reduced TBARS levels. These active and unstable lipid peroxides are formed and can be expressed as equivalents of malondialdehyde (MDA) [33,56]. The kidney and stomach of mice treated with the Mavrodaphne grape stem extract showed decreased TBARS levels, which is in agreement with some previous studies that examined the effects of another by-product of winery industry, i.e., grape pomace, on broilers vital organs [16]. Kidney and stomach of the treated mice revealed decreased GSH levels. These can be affected by a change in the activity of the enzymes that participate in its biosynthetic rate, i.e., γ-glutaminocysteine ligase (γ-GCL) and glutathione synthetase (GS), or by a change in the activity of the enzymes that participate in its recycling rate, i.e., glutathione reductase (GR) and glutathione peroxidase (GPx) [55,57,58]. The regulation of the expression of these enzymes can affect the levels of endogenous GSH [59,60]. Furthermore, TAC levels were found decreased in kidney and stomach of the mice treated with Mavrodaphne grape stem extract. Total antioxidant capacity refers to the ability of tissue antioxidant components to neutralize the excess of free radicals. The main advantage of this biomarker is that it determines the antioxidant capacity of a biological sample as a whole and not just of a single antioxidant [61,62]. Previous literature has demonstrated that the consumption of an animal feed enriched with polyphenolic compounds from grapes enhances the total antioxidant activity in vital organs of farm animals [57,63,64]. To the best of our knowledge this is the first study to investigate the effects of Mavrodaphne grape variety by-products in these species.

Regarding the results obtained after the oral administration of Xinomavro wine extract in rats, significant changes were revealed between the group treated with the wine extract and the untreated group. The rats’ tissues that exhibited the most prominent results were the pancreas, spleen, small intestine, large intestine, testicles, and quadriceps, which showed notably increased GSH levels after the oral administration of the polyphenolic wine extract. Kidney and large intestine tissues revealed high endogenous TAC levels after the oral administration. The administration of the wine extract protected the lungs, liver, large intestine, and testicles against lipid peroxidation. On the contrary, the eyes, stomach, and lungs showed deregulated antioxidant defense mechanisms, supported by the lower TAC levels in the wine extract-treated rats as compared to the untreated. Lower GPx activity was found in the eyes of the experimental group. Brain tissue also revealed a redox imbalance, depicted by the higher lipid peroxidation levels and the lower GR activity in the wine extract-treated rats. Spleen tissue followed the same pattern of increased lipid peroxidation levels and decreased SOD enzyme activity. Kidney tissue showed increased lipid peroxidation levels and increased H_2_O_2_ decomposition rate. Previously, rat kidney showed diminished lipid peroxidation and elevated GSH/GSSG ratio, activity of CAT and GPx during exposure to a wine rich in flavanol for 10 weeks [65]. Grape skin, also a by-product of winery industry, was found to improve antioxidant capacity in rats fed with a high-fat diet, evidenced by the increased serum total antioxidant status and the enzymatic activities of hepatic catalase and superoxide dismutase, xanthine oxidase, and glucose-6-phosphatase [66]. In a similar experimental model, a grapevine leaf extract was also found to induce beneficial effects [67].

Histopathology assessment did not reveal any significant alterations between the treated and the untreated group. These findings are in line with other studies testing polyphenols from extracts of grapes (seeds and peel) [44,45,46] or trans-resveratrol (a specific polyphenol from wine) [56]. To our knowledge, the safety of polyphenolic mixtures contained in wine has not been studied yet. In a 28-day toxicological study in rats with daily oral administration of a polyphenol contained in wine, namely trans-resveratrol, at a dose of 20 mg/kg, no adverse histopathology reactions were present [56].

Furthermore, our results revealed that the oral administration of polyphenols for a duration of 14 days at a dose of 25 mg/kg in rats did not affect the final body weight or the average growth rate. Oral studies of trans-resveratrol (as a component of wine) indicate that no change in body weight or average growth rate was observed compared to the control group [28,29,56]. In addition, Wilson et al. [24] reported that there was no difference in body weight and food consumption between rabbits receiving an hypercholesterolemic diet containing trans-resveratrol and controls. Similarly, sub-chronic studies with extracts from seeds and grape peel have confirmed that there were no significant differences in rat’s growth [44,68]. These results reinforce the findings that polyphenol extract from red wine exerts no effects on growth. During administration, no clinical findings were observed in accordance with studies carried out for longer periods with extracts from individual parts of grapes [43,44,45,46,68,69].

The hematological parameters showed no differences between the treated and untreated groups, and the values were within the reference values proposed for the breed, sex, and age of the examined animals [70]. The ALT and ALP levels, which indicate liver damage, did not differ between groups. The values of the parameters determining renal function (urea and creatinine) did not show a statistically significant difference between the group receiving the extract and the untreated group and were within the reference values. The above findings are similar to studies in which a single polyphenol or extracts from individual parts of grapes have been administered [43,44,45,46,56,68,69].

Examination of vital organs performed during the gross necropsy revealed no changes between the study groups. During histological examination, lesions were observed in tissues taken from lungs in both experimental groups. It should be noted that during the necropsy, there were no apparent changes in the size, color, texture, or composition of the lungs. These lesions were characterized by an increase in perivascular mononuclear cells (mainly lymphocytes), localized around the medium-sized arteries throughout the lungs and extending sometimes to the peribronchiolar areas. In addition, focal infiltrates of mixed inflammatory cells (macrophages, lymphocytes, and occasionally neutrophils) were noted in the median space and in the alveoli. The statistical analysis showed no difference between groups as far as the occurrence and the intensity of the lesions. The dominant presence of lymphocytes refers to chronic inflammation. The above data led us to conclude that the inflammation predated the onset of experimentation and is not related to the administration of the wine extract. Bibliographic data suggest that similar lesions on lungs are common in laboratory animals, at a rate of at least 50% rate, and may be due to several infectious agents [71,72]. In addition, it has been stated that although laboratory animal suppliers guarantee the absence of pathogens, similar lesions on laboratory animals’ lungs have been revealed during sampling examination, before experimentation, which tend to limit themselves as rats mature in age [73,74]. One-year-old rats are mostly free of lesions. The increased presence of the above lesions at the first few weeks of life is probably due to the immature immune system of rats, which, as it matures, explains the tendency for progressive reduction of the observed lesions. Infectious agents have also not been found during histological examination. Based on the above, the dose of 25 mg/kg/day of polyphenols from the administered wine extract appears to be low enough to cause lesions on vital organs.

## 4. Materials and Methods

### 4.1. Chemical Composition and Dose Preparation

In the first experiment, grape stems from Mavrodaphne grape variety were collected from a winery named Ampelones Kolipera in Patras, and the extract was prepared as previously described [50]. The chemical composition of Mavrodaphne grape stem extract was determined using HPLC analysis, and the results were presented thoroughly in the previous publication. Then, the grape stem extract was used for the enrichment of a feeding biscuit, the composition of which is described in Table 5. Based on a phytochemical analysis, the concentration of the grape stem extract corresponded to 32 mg polyphenols/kg body weight/day, a dose previously used by Priftis et al. [55].

In the second experiment, the Xinomavro wine extract variety was used. The wine was condensed into Rotavapor until half its volume was reduced to ensure the complete removal of alcohol naturally contained in wine, since its presence prevents the retention of the polyphenols from the used resins. Then, adsorption polymer resins (Amberlite^®^ XAD-4, Supelco, Bellefonte, PA, USA) were used. For the recovery of the polyphenols, ethanol was used as an organic solvent. Finally, the sample drought in Rotavapor until lyophilization and complete removal of solvents, as previously described [49]. Afterward, the dry weight of the final product was determined. The analysis showed that a bottle of Xinomavro wine (750 mL) contained 3.88 g of phenolics, corresponding to a yield of phenol of 0.51% (per 100 mL of wine). The dose was set at 25 mg of polyphenols per kg of body weight. Since the extract was 26.7% polyphenolic compounds, 34.5 mg of the extract were required for each rat per day (93.2 mg of extract/kg/day). To calculate the doses, the mean body weight value of rats at the beginning of the experiment was used. The doses were prepared by dissolving 34.5 mg of extract in 1.5 mL of distilled water, which was administered daily to the drinking water of rats. Regarding the control group, corresponding doses of 1.5 mL of distilled water were prepared.

### 4.2. Animals and Housing Conditions

In the first experiment, 4-month-old male C57BL/6 mice (25–32 g BW) were housed in the animal care facility of the University of Patras at standard laboratory conditions (12:12 h of light and darkness, free access to food and water) (Permission code: EL-13-BIOexp-04). The protocol was approved by the Research Ethics Committee based on the University’s Code of Ethics and Deontology in Research (Permission code: REC-6207). Mice were randomly divided into two groups (10–12 mice per group): the control (vehicle) and treated group (extract). The grape stem extract of Mavrodaphne was dissolved in 10% dimethyl sulfoxide (DMSO) and 10% cremophor EL in drinking water and was administered orally (gavage), immediately after dissolution, at a dose of 155.9 mg/kg body weight/day for 28 days. Twenty-four hours after the last administration of each feed, animals were anesthetized with isoflurane and sacrificed, and the peripheral tissues were isolated.

In the second experiment, 12 male Wistar rats, aged 20 weeks, were used from Clinical Pharmacology, School of Medicine, Aristotle University of Thessaloniki. Τhe rats were randomly divided into two groups of 6 individuals each. They were housed in cages, in groups of 3, at a temperature of 22 ± 2 °C, with relative humidity of 40–70% and artificial lighting with a 12 h photoperiod of day/night. This was followed by an adjustment period of one week to acclimatize with the members of each cage group. The extract was administered daily at the same time via potable water for 14 days. In one group, 1.5 mL of distilled water was administered with drinking water and served as the control group, while in the second group, 1.5 mL of solution of distilled water and wine extract was administered considered as test group. During the experiment, water, and dry food (pellets) intended for laboratory animals were provided daily and ad libitum. Each animal was labeled with an ecological color on the coat to be identified. The experiment was performed in the animal facility of the laboratory of Clinical Pharmacology, School of Medicine, Aristotle University of Thessaloniki in accordance with the Helsinki Declaration and National standards (Permission code: EL-54-BIOexp-04). The experimental protocol was approved by the National Veterinary Administration authorities (License No.: 47667(198)). All animals were treated in accordance with the guiding principles of the European Community Council Directive (89/609/EEC) for the care and use of laboratory animals. At the end of the experiment, the rats were fasted overnight and anesthetized with isoflurane.

### 4.3. Redox Biomarkers

#### 4.3.1. Tissue Homogenization

Tissue samples were homogenized using a Minilys homogenizer (Bertin Instruments, Montigny-le-Bretonneux, France). Briefly, into homogenization tubes, 200–250 mg of each tissue and 600–750 μL of a solution comprising phosphate-buffered saline (PBS) and protease inhibitors (Complete^TM^ mini protease inhibitors, Roche Diagnostics, Mannheim, Germany) were added and homogenized for 30 sec at the highest available speed. A centrifugation (15,000× *g*, 5 min, 4 °C) followed, and the supernatant was collected, separated in aliquots, stored at −80 °C, and thawed only once before each analysis. Total protein concentration was determined using the Bradford assay, as previously described [75].

Each assay was performed in triplicate and within 3 months of blood collection. Blood samples were aliquoted at −80 °C and thawed once before the analysis.

#### 4.3.2. Determination of GSH

GSH levels were determined using a slightly modified version of the method of Reddy et al. [76], as previously described by Veskoukis et al. [77]. At first, 200 μL red blood cell lysate (RBCL) or 100 μL tissue homogenate were mixed with 5% trichloroacetic acid (TCA). The samples were centrifuged (15,000× *g*, 5 min, 5 °C), and the supernatant was transferred to a new microcentrifuge tube. Regarding RBCL, 150 μL of the supernatant were mixed with 45 μL 5% TCA and centrifuged (15,000× *g*, 5 min, 5 °C). After that, in both cases, 20 μL of the supernatant was mixed with 660 μL sodium potassium phosphate buffer (67 mM, pH = 7.95) and 330 μL 5,5′-dithiobis-2 nitrobenzoate (DTNB) (1 mM). Then, an incubation took place in dark at room temperature for 15 min, and the optical density was measured at 412 nm.

#### 4.3.3. Determination of H_2_O_2_ Decomposition Rate

A slightly modified version of the method of Aebi [78], as previously described by Veskoukis et al. [77], was used for the determination of H_2_O_2_ decomposition rate. The mixture contained 4 μL RBCL (diluted 1:10) or 5 μL tissue homogenate, and 2991 or 2990 μL of sodium potassium phosphate buffer (67 mM, pH = 7.4), respectively. Then, an incubation took place at 37 °C for 10 min. Afterward, 5 μL 30% H_2_O_2_ was added to the samples. The change in optical density was immediately monitored at 240 nm for 130 s. The H_2_O_2_ decomposition rate was calculated based on the molar extinction coefficient of H_2_O_2_ (43.6 M^−1^ cm^−1^).

#### 4.3.4. Determination of TAC

The determination of TAC levels was based on the method of Janaszewska and Bartosz [79]. The reaction mixture contained 40 μL tissue homogenate or 20 μL plasma; 460 or 480 μL phosphate buffer (10 mM, pH = 7.4), respectively; and 500 μL 2,2-diphenyl-1-picrylhydrazyl radical (DPPH^•^) (0.1 mM) solution. An incubation took place in dark at room temperature for 60 min. Finally, the samples were centrifuged (15,000× *g*, 3 min, 25 °C), and the optical density was measured at 520 nm.

#### 4.3.5. Determination of TBARS

A slightly modified version of the assay of Keles et al. [80] was used for TBARS determination, as previously described by Spanidis et al. [81]. Specifically, 100 μL plasma or tissue homogenate was added to 500 μL 35% TCA and 500 μL Tris–HCl (pH = 7.4). An incubation took place at room temperature for 10 min. Then, 1 mL of a solution containing sodium sulphate (Na_2_SO_4_) (2 M) and thiobarbituric acid (TBA) (55 mM) was added and the samples were incubated for 45 min at 95 °C. Then, the samples were transferred at 4 °C for 5 min, followed by the addition of 1 mL 70% TCA. Afterward, 1 mL from each sample was centrifuged (11,200× *g*, 3 min, 25 °C) and the optical density was measured at 530 nm. The calculation of TBARS levels was based on the molar extinction coefficient of malondialdehyde (155 × 10^3^ M^−1^cm^−1^).

#### 4.3.6. Determination of Protein Carbonyls

A slightly modified version of the method of Patsoukis et al. [82] was used for protein carbonyls determination, as previously described by Veskoukis et al. [77]. Initially, 50 μL 20% TCA was added to 50 μL plasma or tissue homogenate. An incubation took place for 15 min at 4 °C, followed by centrifugation (15,000× *g*, 5 min, 4 °C). Subsequently, the supernatant was discarded, and the pellet was resuspended in 500 μL 2,4-dinitrophenylhydrazine (DNPH) (10 mM) (diluted in 2.5 N HCl). A blank for each sample containing the same sample volume was resuspended with 500 μL HCl (2.5 N). The samples and their respective blanks were incubated in the dark at room temperature for 1 h with intermittent vortexing every 15 min, followed by centrifugation (15,000× *g*, 5 min, 4 °C). After centrifugation, the supernatant was discarded, and the pellets were resuspended with 1 mL 10% TCA. Afterward, the samples and blanks were centrifuged again (15,000× *g*, 5 min, 4 °C), and the pellets were washed three times with 1 mL of an ethanol–ethyl acetate mixture (1:1 *v*/*v*). The pellets were resuspended, and the samples and blanks were centrifuged (15,000× *g*, 5 min, 4 °C). After the third wash and the removal of the supernatant, the pellets were resuspended with 1 mL urea (5 M, pH = 2.3), vortexed, and incubated at 37 °C for 15 min. Finally, the samples were centrifuged (15,000× *g*, 5 min, 4 °C), and their optical density was determined at 375 nm. The calculation of the protein carbonyls concentration was based on the molar extinction coefficient of DNPH (22 × 10^3^ M^−1^cm^−1^).

#### 4.3.7. Determination of SOD Activity

The determination of SOD activity was based on the method of nitroblue tetrazolium salt (NBT), as described by Oberley and Spitz [83]. In the reaction mixture, 100 μL of RBCL (diluted 1:100) and 800 μL of a master mix (diethylenetriaminepentaacetic acid (DETAPAC) (1.34 mM), NBT (2.24 mM), and xanthine (1.18 mM)) were added, followed by the addition of xanthine oxidase (~60 mU). The optical density was measured at 560 nm for 1.5 min. SOD activity was calculated based on the percentage inhibition of NBT.

#### 4.3.8. Determination of GPx Activity

Glutathione Peroxidase (GPx) activity was measured according to Flohe and Gunzler [84], as previously described by Veskoukis et al. [77]. Briefly, 500 μL of phosphate buffer (100 mM, pH = 7), 100 μL of glutathione reductase (GR) (0.24 U), 100 μL of GSH (10 mM), and 100 μL of RBCL (diluted 1:100) were added to a microcentrifuge tube. The mixture was incubated for 10 min at room temperature. Then, 100 μL of nicotinamide adenine dinucleotide phosphate (NADPH) (1.5 mM) in 0.1% NaHCO_3_ solution was added, followed the transfer of the mixture in a cuvette. An incubation took place for 3 min at room temperature. The reaction was started by adding 100 μL of tert-Butyl hydroperoxide (t-BuOOH) (12 mM). The decrease in optical density was monitored at 340 nm for 5 min. The calculation of GPx activity was based on the molar extinction coefficient of NADPH (6200 L/mol/cm).

#### 4.3.9. Determination of GR Activity

Glutathione reductase (GR) activity was measured according to the protocol of Smith et al. [85], described previously by Veskoukis et al. [77]. The reaction mixture contained 700 μL of phosphate buffer (200 mM, 1 mM ethylenediaminetetraacetic acid (EDTA), pH = 7.5), 250 μL of DTNB (3 mM) in phosphate buffer (200 mM), 50 μL of β-NADPH (2 mM) in phosphate buffer (200 mM), and 50 μL of GSSG (20 mM) in phosphate buffer (200 mM). The reaction started following the addition of 25 μL RBCL (diluted 1:20). The change in optical density was monitored at 412 nm for 1 min.

### 4.4. Body Weight and Growth Rate

Body weight was measured on the 1st, 7th, and last day of administration before euthanasia. Food and water consumption were recorded daily. The growth rate was calculated as the difference between the final weight and the initial weight divided by the days of administration. During administration, the behavior of experimental animals inside and outside of the cage was checked, and clinical examination was performed. Furthermore, 12 h after administration, the experimental animals were checked for mortality. In case of severe disturbance of welfare, which was not expected from the bibliographical data, euthanasia and autopsy were performed. All handlings took place at the same time every morning.

### 4.5. Hematological and Clinical Chemistry Parameters

At the end of the study, the rats were fasted overnight and anesthetized with isoflurane. Blood was collected by cardiac puncture from the right ventricle (1 mL in a blood collection vial with EDTA for hematology and 2 mL in a vial with sodium heparin for blood clinical chemistry). The blood was centrifuged (1500 rpm, 15 min, 4 °C) for the separation of erythrocytes and plasma. All samples were processed within 4–6 h in the hematology and clinical chemistry laboratory of the Faculty of Veterinary Medicine of the University of Thessaloniki.

The following hematological parameters were determined with a ADVIA120 (Siemens Healthineers) hematological analyzer: the number of red blood cells, hemoglobin, hematocrit, mean corpuscular volume, mean corpuscular hemoglobin content, mean corpuscular hemoglobin concentration, total white blood cell count, leukocyte type, platelet count, and average platelet volume. The following clinical chemistry parameters were also determined with a Vitalab Flexor E analyzer (VitalScientific N.V., Dieren, The Netherlands): ALT, ALP, total proteins, albumin, creatinine, urea, GGT.

### 4.6. Gross Necropsy

After blood collection, rats remained under anesthesia and were euthanized by dislocation of the first cervical vertebra. A detailed gross necropsy was carried out, including careful examination of the external body (skin, hair, mucous membranes); the oral and nasal cavities; the anus; the external genitalia; the cranial, thoracic, and abdominal cavities; and the internal organs. Lungs, spleen, heart, liver, kidneys, stomach, the entire intestinal tract (duodenum, nitistida, ileus, colon), pancreas, and testicles were collected and trimmed from any adherent tissue. The aforementioned organs were evaluated in terms of size, color, and texture. Any lesions were described in detail. The inner content of hollow organs and cavities was also examined. The results of the necropsy were recorded.

### 4.7. Histopathology

The tissues were stabilized in a 10% neutral pH formalin solution, treated with graded alcohol, embedded in paraffin, sectioned in 5 μm slices, and stained with hematoxylin/eosin. The tissues were examined under the light of microscope (Nikon Eclipse E400, Melville, NY, USA), and the architecture, cell degeneration/necrosis, inflammatory filtration, hemodynamic lesions, and cell growth disorders were evaluated. The intensity of the findings was categorized as minimal, mild, moderate, and significant. The results were recorded.

### 4.8. Statistical Analyses

The results of histopathological analysis are expressed as mean ± standard deviation (SD). For quantitative data, the Kolmogorov–Smirnov test was performed to check if variables followed normal distribution. Levene’s test was performed to check for homogeneity of variances. In the case of abnormal distribution, data were analyzed with the non-parametric Kruskal–Wallis test. The Chi-square test (χ^2^ test) and Fisher’s exact test were performed for quality variables. The IBM SPSS Statistics version 26.0 was used to analyze the data. The significance level was set at a *p*-value less than 0.05 (*p* < 0.05).

The results of redox biomarkers are expressed as mean ± standard error of the mean (SEM). The Shapiro–Wilk test was performed to check if the variables followed a normal distribution. In the case of abnormal distribution, the data were analyzed with the non-parametric Kruskal–Wallis test. The GraphPad Prism version 8.0.0 was used to analyze the data. The significance level was set at a *p*-value less than 0.05 (*p* < 0.05).

## 5. Conclusions

The present study investigated the biological effects of a wine and a grape stem extract derived from the emblematic Greek red grape varieties Xinomavro and Mavrodaphne, respectively. According to our results, Xinomavro wine extract did not exert any effects on body weight, growth rate, clinical, biochemical, and histopathological parameters; however, it affected rat redox homeostasis by inducing beneficial and harmful outcomes depending on the examined tissue. More specifically, in some tissues, Xinomavro wine extract exerted antioxidant effects by enhancing the antioxidant arsenal and by preventing from the detrimental effects of oxidative damage, whereas in other tissues, it exerted prooxidant effects by deregulating the antioxidant defense mechanisms and by promoting lipid peroxidation. This phenomenon is indicative of the complexity that characterizes the biological systems, overturning the perception of the putative unified action of polyphenol-rich extracts upon administration to living organisms. Contrariwise, the results obtained from the determination of the effects of Mavrodaphne grape stem extract on mice redox biomarkers clearly demonstrated that the extract exerted beneficial effects by preventing from the promotion of lipid peroxidation, while the alterations induced in the antioxidant defense mechanisms could be considered as redox adaptations. This study provides valuable insights into the biological activity of the polyphenol-rich Mavrodaphne grape stem extract that could be utilized in the development of bio-functional animal feeds, thus improving animal redox and health status, while the exploitation of these compounds constitutes an ecofriendly management of by-products produced during the vinification process.

## Figures and Tables

**Table 1:** Results of the analyses of rat blood parameters. Values are expressed as mean ± SD.

Blood Parameters (Unit)	Control Group *n* = 6	Extract Group *n* = 6
Red blood cells (RBC)(M/μL)	8.63 ± 0.45	8.42 ± 0.21
Hemoglobin (HGB) (g/dL)	15.97 ± 0.75	15.46 ± 0.58
Μean corpuscular volume volume (MCV) (fl)	50.63 ± 0.79	50.64 ± 1.51
Mean corpuscular hemoglobin (MCH) (pg)	16.58 ± 0.15	16.42 ± 0.83
Mean corpuscular hemoglobin concentration (MCHC) (g/dL)	32.78 ± 0.60	32.40 ± 0.72
Hematocrit (HCT) (%)	44.78 ± 2.94	45.68 ± 1.19
White blood cells (WBC)(K/μL)	5.78 ± 0.92	5.34 ± 2.34
Neutrophils (K/μL %)	19.77 ± 2.74	26.24 ± 9.82
Lymphocytes (K/μL %	75.67 ± 2.52	70.10 ± 9.99
Monocytes (K/μL %)	2.37 ± 0.23	2.08 ± 0.34
Eosinophils (K/μL %)	1.37 ± 0.60	0.86 ± 0.38
Basophils (K/μL %)	0.70 ± 0.15	0.62 ± 0.08
Platelets (PLT) (K/μL)	656.33 ± 65.48	645.80 ± 102.47
Μean platelets volume (MPV) (fl)	7.62 ± 0.56	8.34 ± 0.56

**Table 2:** Results of the analyses of rat clinical chemistry parameters. Values are expressed as mean ± SD.

Blood Serum Results	Control Group *n* = 6	Extract Group *n* = 6
Total proteins (g/dL)	6.7 ± 0.21	6.68 ± 0.23
Albumin (g/mL)	4.32 ± 0.12	4.3 ± 0.10
Urea (mg/mL)	15.17 ± 4.96	14.33 ± 5.86
Creatinine (mg/mL)	0.6 ± 0	0.47 ± 0.06
Alanine-aminotransferase (ALT) (U/lit)	200.33 ± 11.5	250.67 ± 53.45
Alkaline phosphatase (ALP) (U/lit)	53.17 ± 5.27	51 ± 1.41
Gamma-glutamyl transferase (GGT) (U/lit)	0	0

**Table 3:** Histology type of lesions in lungs.

Lung Type of Lesions	Control Group *n* = 6	Extract Group *n* = 6
Architecture	- ^1^	-
Cell degeneration/necrosis	-	-
Inflammatory filtration	4 ^2^	4
Hemodynamic damage	-	-
Cell growth disorders	-	-

^1^ Lack of lesions; ^2^ number of rats with specific type of lesions.

**Table 4:** Severity of lesions in lungs.

Lung Severity	Control Group *n* = 4	Extract Group *n* = 4
Minimum	-	-
Mild	3 ^1^	2
Medium	1 ^2^	2
Serious	-	-

^1^ Number of rats with mild lesions; ^2^ number of rats with medium lesions.

**Table 5:** The composition of the enriched feeding biscuit.

A/A	Ingredients	Quantity	
1	Whey protein 80% (sheep goat)	4000	kg
2	Debatted cocoa powder	3500	kg
3	Coconut sugar	3000	kg
4	Pasteurized egg white	2600	kg
5	Olive oil	2400	lt
6	Coconut oil	2300	lt
7	Carob flour	2000	kg
8	Carob honey	1500	kg
9	Grape stem extract	0.155	kg
10	Sodium bicarbonate	0.150	kg
	Total	21,605	

## Data Availability

All data are available upon request from the corresponding author.

## Sample Availability

Samples of the compounds are available from the authors.

## References

[1] Giovannelli L., Testa G., De Filippo C., Cheynier V., Clifford M., Dolara P. (2000). Effect of complex polyphenols and tannins from red wine on DNA oxidative damage of rat colon mucosa in vivo. Eur. J. Nutr..

[2] Facinó R., Carini M., Aldini G., Berti F., Rossoni G., Bombardelli E., Morazzoni P. (1996). Procyanidines from *Vitis vinifera* Seeds Protect Rabbit Heart from Ischemia/Reperfusion Injury: Antioxidant Intervention and/or Iron and Copper Sequestering Ability. Planta Med..

[3] Bagchi D., Garg A., Krohn R.L., Bagchi M., Tran M.X., Stohs S.J. (1997). Oxygen free radical scavenging abilities of vitamins C and E, and a grape seed proanthocyanidin extract in vitro. Res. Commun. Mol. Pathol. Pharmacol..

[4] Bagchi D., Garg A., Krohn R.L., Bagchi M., Bagchi D.J., Balmoori J., Stohs S.J. (1998). Protective Effects of Grape Seed Proanthocyanidins and Selected Antioxidants against TPA-Induced Hepatic and Brain Lipid Peroxidation and DNA Fragmentation, and Peritoneal Macrophage Activation in Mice. Gen. Pharmacol..

[5] Zafirov D., Bredy-Dobreva G., Litchev V., Papasova M. (1990). Antiexudative and capillaritonic effects of procyanidines isolated from grape seeds (*V. vinifera*). Acta Physiol. Pharmacol. Bulg..

[6] Frankel E.N., Waterhouse A.L., Teissedre P.L. (1995). Principal Phenolic Phytochemicals in Selected California Wines and Their Antioxidant Activity in Inhibiting Oxidation of Human Low-Density Lipoproteins. J. Agric. Food Chem..

[7] Rice-Evans C., Miller N., Paganga G. (1997). Antioxidant properties of phenolic compounds. Trends Plant Sci..

[8] Rice-Evans C.A., Miller N.J. (1996). Antioxidant activities of flavonoids as bioactive components of food. Biochem. Soc. Trans..

[9] Salucci S., Burattini S., Giordano F.M., Lucarini S., Diamantini G., Falcieri E. (2017). Further Highlighting on the Prevention of Oxidative Damage by Polyphenol-Rich Wine Extracts. J. Med. Food.

[10] Fauconneau B., Waffo-Teguo P., Huguet F., Barrier L., Decendit A., Merillon J.-M. (1997). Comparative study of radical scavenger and antioxidant properties of phenolic compounds from Vitis vinifera cell cultures using in vitro tests. Life Sci..

[11] Biasi F., Deiana M., Guina T., Gamba P., Leonarduzzi G., Poli G. (2014). Wine consumption and intestinal redox homeostasis. Redox Biol..

[12] Dolara P., Luceri C., De Filippo C., Femia A.P., Giovannelli L., Caderni G., Cecchini C., Silvi S., Orpianesi C., Cresci A. (2005). Red wine polyphenols influence carcinogenesis, intestinal microflora, oxidative damage and gene expression profiles of colonic mucosa in F344 rats. Mutat. Res. Mol. Mech. Mutagen..

[13] Rodrigo R., Miranda A., Vergara L. (2011). Modulation of endogenous antioxidant system by wine polyphenols in human disease. Clin. Chim. Acta.

[14] Nunes C., Ferreira E., Freitas V., Almeida L., Barbosa R.M., Laranjinha J. (2013). Intestinal anti-inflammatory activity of red wine extract: Unveiling the mechanisms in colonic epithelial cells. Food Funct..

[15] Chen C.K., Pace-Asciak C.R. (1996). Vasorelaxing activity of resveratrol and quercetin in isolated rat aorta. Gen. Pharmacol. Vasc. Syst..

[16] Basli A., Soulet S., Chaher N., Mérillon J.-M., Chibane M., Monti J.-P., Richard T. (2012). Wine Polyphenols: Potential Agents in Neuroprotection. Oxidative Med. Cell. Longev..

[17] Caimi G., Carollo C., Presti R.L. (2003). Wine and endothelial function. Drugs Under Exp. Clin. Res..

[18] Castaldo L., Narváez A., Izzo L., Graziani G., Gaspari A., Di Minno G., Ritieni A. (2019). Red Wine Consumption and Cardiovascular Health. Molecules.

[19] Rasines-Perea Z., Teissedre P.-L. (2017). Grape Polyphenols’ Effects in Human Cardiovascular Diseases and Diabetes. Molecules.

[20] Gresele P., Cerletti C., Guglielmini G., Pignatelli P., de Gaetano G., Violi F. (2011). Effects of resveratrol and other wine polyphenols on vascular function: An update. J. Nutr. Biochem..

[21] Riccioni G., Gammone M.A., Tettamanti G., Bergante S., Pluchinotta F.R., D’Orazio N. (2015). Resveratrol and anti-atherogenic effects. Int. J. Food Sci. Nutr..

[22] Babál P., Kristová V., Černá A., Janega P., Pecháňová O., Danihel L., Andriantsitohaina R. (2006). Red wine polyphenols prevent endothelial damage induced by CCl4 administration. Physiol. Res..

[23] Johnson W., Morrissey R., Usborne A., Kapetanovic I., Crowell J., Muzzio M., McCormick D. (2011). Subchronic oral toxicity and cardiovascular safety pharmacology studies of resveratrol, a naturally occurring polyphenol with cancer preventive activity. Food Chem. Toxicol..

[24] Wilson T., Knight T.J., Beitz D.C., Lewis D.S., Engen R.L. (1996). Resveratrol promotes atherosclerosis in hypercholesterolemic rabbits. Life Sci..

[25] Bertelli A.A., Giovannini L., Giannessi D., Migliori M., Bernini W., Fregoni M. (1995). Antiplatelet activity of synthetic and natural resveratrol in red wine. Int. J. Tissue React..

[26] Jang M., Cai L., Udeani G.O., Slowing K.V., Thomas C.F., Beecher C.W.W., Fong H.H.S., Farnsworth N.R., Kinghorn A.D., Mehta R.G. (1997). Cancer Chemopreventive Activity of Resveratrol, a Natural Product Derived from Grapes. Science.

[27] Turner R.T., Evans G.L., Zhang M., Maran A., Sibonga J.D. (1999). Is Resveratrol an Estrogen Agonist in Growing Rats?. Endocrinology.

[28] Carbó N., Costelli P., Baccino F.M., López-Soriano F.J., Argilés J.M. (1999). Resveratrol, a Natural Product Present in Wine, Decreases Tumour Growth in a Rat Tumour Model. Biochem. Biophys. Res. Commun..

[29] Turrens J.F., Lariccia J., Nair M.G. (1997). Resveratrol Has No Effect on Lipoprotein Profile and Does Not Prevent Peroxidation of Serum Lipids in Normal Rats. Free. Radic. Res..

[30] Halpern M.J., Dahlgren A.-L., Laakso I., Seppänen-Laakso T., Dahlgren J., McAnulty P.A. (1998). Red-wine Polyphenols and Inhibition of Platelet Aggregation: Possible Mechanisms, and Potential Use in Health Promotion and Disease Prevention. J. Int. Med. Res..

[31] Zhao J., Wang J., Chen Y., Agarwal R. (1999). Anti-tumor-promoting activity of a polyphenolic fraction isolated from grape seeds in the mouse skin two-stage initiation–promotion protocol and identification of procyanidin B5-3′-gallate as the most effective antioxidant constituent. Carcinog..

[32] Velmurugan B., Singh R.P., Kaul N., Agarwal R., Agarwal C. (2010). Dietary Feeding of Grape Seed Extract Prevents Intestinal Tumorigenesis in APCmin/+ Mice. Neoplasia.

[33] Waterhouse A.L. (2002). Wine Phenolics. Ann. N. Y. Acad. Sci..

[34] Saric S., Sivamani R.K. (2016). Polyphenols and Sunburn. Int. J. Mol. Sci..

[35] Katiyar S.K., Afaq F., Azizuddin K., Mukhtar H. (2001). Inhibition of UVB-Induced Oxidative Stress-Mediated Phosphorylation of Mitogen-Activated Protein Kinase Signaling Pathways in Cultured Human Epidermal Keratinocytes by Green Tea Polyphenol (−)-Epigallocatechin-3-gallate. Toxicol. Appl. Pharmacol..

[36] Filip A., Daicoviciu D., Clichici S., Bolfa P., Catoi C., Baldea I., Bolojan L., Olteanu D., Muresan A., Postescu I. (2011). The effects of grape seeds polyphenols on SKH-1 mice skin irradiated with multiple doses of UV-B. J. Photochem. Photobiol. B Biol..

[37] Bouhamidi R., Prévost V., Nouvelot A. (1998). High protection by grape seed proanthocyanidins (GSPC) of polyunsaturated fatty acids against UV-C induced peroxidation. C. R. Acad. Sci. III.

[38] Choi J., Ryu S.-J., Kim K.-J., Kim H.-M., Chung H.-C., Lee B.-Y. (2018). Single, 14-Day, and 13-Week Repeated Dose Toxicity Studies of Daily Oral Gelidium elegans Extract Administration to Rats. Molecules.

[39] Vijayasteltar L., Nair G.G., Maliakel B., Kuttan R., Krishnakumar I.M. (2016). Safety assessment of a standardized polyphenolic extract of clove buds: Subchronic toxicity and mutagenicity studies. Toxicol. Rep..

[40] Cladis D.P., Li S., Reddivari L., Cox A., Ferruzzi M.G., Weaver C.M. (2020). A 90 day oral toxicity study of blueberry polyphenols in ovariectomized sprague-dawley rats. Food Chem. Toxicol..

[41] Tanaka W., Yokoyama D., Matsuura Y., Nozaki M., Hirozawa N., Kunitake H., Sakono M., Sakakibara H. (2019). Subchronic toxicity evaluation of leaves from rabbiteye blueberry (*Vaccinium virgatum* Aiton) in rats. Toxicol. Rep..

[42] Takami S., Imai T., Hasumura M., Cho Y.-M., Onose J., Hirose M. (2008). Evaluation of toxicity of green tea catechins with 90-day dietary administration to F344 rats. Food Chem. Toxicol..

[43] Lluís L., Muñoz M., Nogués M.R., Sánchez-Martos V., Romeu M., Giralt M., Valls J., Solà R. (2011). Toxicology evaluation of a procyanidin-rich extract from grape skins and seeds. Food Chem. Toxicol..

[44] Inoue K., Morikawa T., Takahashi M., Yoshida M., Ogawa K. (2013). A 13-week subchronic toxicity study of grape skin extract in F344 rats. J. Toxicol. Sci..

[45] Yamakoshi J., Saito M., Kataoka S., Kikuchi M. (2002). Safety evaluation of proanthocyanidin-rich extract from grape seeds. Food Chem. Toxicol..

[46] Bentivegna S., Whitney K. (2002). Subchronic 3-month oral toxicity study of grape seed and grape skin extracts. Food Chem. Toxicol..

[47] Rodriguez-Lopez P., Rueda-Robles A., Borrás-Linares I., Quirantes-Piné R.M., Emanuelli T., Segura-Carretero A., Lozano-Sánchez J. (2022). Grape and Grape-Based Product Polyphenols: A Systematic Review of Health Properties, Bioavailability, and Gut Microbiota Interactions. Horticulturae.

[48] Giovinazzo G., Carluccio M.A., Grieco F. (2019). Wine Polyphenols and Health. Bioactive Molecules in Food.

[49] Tekos F., Makri S., Skaperda Z.-V., Patouna A., Terizi K., Kyriazis I., Kotseridis Y., Mikropoulou E., Papaefstathiou G., Halabalaki M. (2021). Assessment of Antioxidant and Antimutagenic Properties of Red and White Wine Extracts In Vitro. Metabolites.

[50] Veskoukis A.S., Vassi E., Poulas K., Kokkinakis E., Asprodini E., Haroutounian S., Kouretas D. (2020). Grape Stem Extracts From Three Native Greek Vine Varieties Exhibit Strong Antioxidant and Antimutagenic Properties. Anticancer. Res..

[51] Teixeira A., Baenas N., Dominguez-Perles R., Barros A., Rosa E., Moreno D.A., Garcia-Viguera C. (2014). Natural Bioactive Compounds from Winery By-Products as Health Promoters: A Review. Int. J. Mol. Sci..

[52] Troncozo M.I., Lješević M., Beškoski V.P., Anđelković B., Balatti P.A., Saparrat M.C. (2019). Fungal transformation and reduction of phytotoxicity of grape pomace waste. Chemosphere.

[53] Tapia-Quirós P., Montenegro-Landívar M.F., Reig M., Vecino X., Cortina J.L., Saurina J., Granados M. (2022). Recovery of Polyphenols from Agri-Food By-Products: The Olive Oil and Winery Industries Cases. Foods.

[54] Vassi E., Veskoukis A.S., Tekos F., Skaperda Z., Poulas K., Haroutounian S., Kouretas D. (2022). Biological effects of grape stem extracts on human cancer cell lines. Int. J. Funct. Nutr..

[55] Priftis A., Soursou V., Makiou A.-S., Tekos F., Veskoukis A.S., Tsantarliotou M.P., Taitzoglou I.A., Kouretas D. (2019). A lightly roasted coffee extract improves blood and tissue redox status in rats through enhancement of GSH biosynthesis. Food Chem. Toxicol..

[56] Juan M.E., Vinardell M.P., Planas J.M. (2002). The Daily Oral Administration of High Doses of trans-Resveratrol to Rats for 28 Days Is Not Harmful. J. Nutr..

[57] Makri S., Kafantaris I., Stagos D., Chamokeridou T., Petrotos K., Gerasopoulos K., Mpesios A., Goutzourelas N., Kokkas S., Goulas P. (2017). Novel feed including bioactive compounds from winery wastes improved broilers’ redox status in blood and tissues of vital organs. Food Chem. Toxicol..

[58] Aquilano K., Baldelli S., Ciriolo M.R. (2014). Glutathione: New roles in redox signaling for an old antioxidant. Front. Pharmacol..

[59] Kumar H., Kim I.-S., More S.V., Kim B.-W., Choi D.-K. (2014). Natural product-derived pharmacological modulators of Nrf2/ARE pathway for chronic diseases. Nat. Prod. Rep..

[60] Rahal A., Kumar A., Singh V., Yadav B., Tiwari R., Chakraborty S., Dhama K. (2014). Oxidative Stress, Prooxidants, and Antioxidants: The Interplay. BioMed Res. Int..

[61] Skaperda Z., Argyriadou A., Nechalioti P., Alvanou M., Makri S., Bouroutzika E., Kyriazis I., Tekos F., Veskoukis A., Kallitsis T. (2021). Redox Biomarker Baseline Levels in Cattle Tissues and Their Relationships with Meat Quality. Antioxidants.

[62] Kusano C., Ferrari B. (2008). Total Antioxidant Capacity: A Biomarker in Biomedical and Nutritional Studies. J. Cell Mol. Biol..

[63] Kafantaris I., Stagos D., Kotsampasi B., Hatzis A., Kypriotakis A., Gerasopoulos K., Makri S., Goutzourelas N., Mitsagga C., Giavasis I. (2018). Grape pomace improves performance, antioxidant status, fecal microbiota and meat quality of piglets. Animal.

[64] Kerasioti E., Terzopoulou Z., Komini O., Kafantaris I., Makri S., Stagos D., Gerasopoulos K., Anisimov N.Y., Tsatsakis A.M., Kouretas D. (2017). Tissue specific effects of feeds supplemented with grape pomace or olive oil mill wastewater on detoxification enzymes in sheep. Toxicol. Rep..

[65] Rodrigo R., Rivera G., Orellana M., Araya J., Bosco C. (2002). Rat kidney antioxidant response to long-term exposure to flavonol rich red wine. Life Sci..

[66] Lee S.-J., Choi S.-K., Seo J.-S. (2009). Grape skin improves antioxidant capacity in rats fed a high fat diet. Nutr. Res. Pract..

[67] Yu Q.-M., Lim E.-J., Choi S.-K., Seo J.-S. (2014). Antioxidant effect of grapevine leaf extract on the oxidative stress induced by a high-fat diet in rats. Food Sci. Biotechnol..

[68] Ray S., Bagchi D., Lim P.M., Bagchi M., Gross S.M., Kothari S.C., Preuss H.G., Stohs S.J. (2001). Acute and long-term safety evaluation of a novel IH636 grape seed proanthocyanidin extract. Res. Commun. Mol. Pathol. Pharmacol..

[69] Wren A.F., Cleary M., Frantz C., Melton S., Norris L. (2002). 90-Day Oral Toxicity Study of a Grape Seed Extract (IH636) in Rats. J. Agric. Food Chem..

[70] Filho W.J., Lima C.C., Paunksnis M.R.R., Silva A.A., Perilhão M.S., Caldeira M., Bocalini D., De Souza R.R. (2018). Reference database of hematological parameters for growing and aging rats. Aging Male.

[71] Albers T.M., Simon M.A., Clifford C.B. (2009). Histopathology of Naturally Transmitted “Rat Respiratory Virus”: Progression of Lesions and Proposed Diagnostic Criteria. Vet. Pathol..

[72] Baker D.G. (1998). Natural Pathogens of Laboratory Mice, Rats, and Rabbits and Their Effects on Research. Clin. Microbiol. Rev..

[73] Slaoui M., Dreef H.C., Van Esch E. (1998). Inflammatory Lesions in the Lungs of Wistar Rats. Toxicol. Pathol..

[74] Elwell M.R., Mahler J.F., Rao G. (1997). “Have You Seen This?” Inflammatory Lesions in the Lungs of Rats. Toxicol. Pathol..

[75] Bradford M.M. (1976). A rapid and sensitive method for the quantitation of microgram quantities of protein utilizing the principle of protein-dye binding. Anal. Biochem..

[76] Reddy Y.N., Murthy S.V., Dr K., Prabhakar M.C. (2004). Role of Free Radicals and Antioxidants in Tuberculosis Patients. Indian J. Tuberc..

[77] Veskoukis A.S., Kyparos A., Paschalis V., Nikolaidis M.G. (2016). Spectrophotometric assays for measuring redox biomarkers in blood. Biomarkers.

[78] Aebi H. (1984). Catalase in vitro. Oxygen Radicals in Biological Systems.

[79] Janaszewska A., Bartosz G. (2002). Assay of total antioxidant capacity: Comparison of four methods as applied to human blood plasma. Scand. J. Clin. Lab. Investig..

[80] Keles M., Taysi S., Sen N., Aksoy H., Akçay F. (2001). Effect of Corticosteroid Therapy on Serum and CSF Malondialdehyde and Antioxidant Proteins in Multiple Sclerosis. Can. J. Neurol. Sci. J. Can. Sci. Neurol..

[81] Spanidis Y., Goutzourelas N., Stagos D., Mpesios A., Priftis A., Bar-Or D., Spandidos D.A., Tsatsakis A.M., Leon G., Kouretas D. (2016). Variations in oxidative stress markers in elite basketball players at the beginning and end of a season. Exp. Ther. Med..

[82] Patsoukis N., Zervoudakis G., Panagopoulos N.T., Georgiou C.D., Angelatou F., Matsokis N.A. (2004). Thiol redox state (TRS) and oxidative stress in the mouse hippocampus after pentylenetetrazol-induced epileptic seizure. Neurosci. Lett..

[83] Oberley L.W., Spitz D.R. (1984). Assay of superoxide dismutase activity in tumor tissue. Methods Enzymol..

[84] Flohé L., Günzler W.A. (1984). Assays of glutathione peroxidase. Methods Enzymol..

[85] Smith I.K., Vierheller T.L., Thorne C.A. (1988). Assay of glutathione reductase in crude tissue homogenates using 5,5′-dithiobis(2-nitrobenzoic acid). Anal. Biochem..

